# Epstein–Barr Virus, but Not Cytomegalovirus, Latency Accelerates the Decay of Childhood Measles and Rubella Vaccine Responses—A 10-Year Follow-up of a Swedish Birth Cohort

**DOI:** 10.3389/fimmu.2017.01865

**Published:** 2017-12-21

**Authors:** Gintare Lasaviciute, Sophia Björkander, Claudia Carvalho-Queiroz, Ida Hed Myrberg, Bianca Nussbaum, Caroline Nilsson, Mats Bemark, Anna Nilsson, Eva Sverremark-Ekström, Shanie Saghafian-Hedengren

**Affiliations:** ^1^Department of Molecular Biosciences, The Wenner-Gren Institute, Stockholm University, Stockholm, Sweden; ^2^Department of Women’s and Children’s Health, Childhood Cancer Research Unit, Astrid Lindgren Children’s Hospital, Karolinska Institutet, Stockholm, Sweden; ^3^Department of Clinical Science and Education, Södersjukhuset, Karolinska Institutet and Sachs’ Children and Youth Hospital, Stockholm, Sweden; ^4^Department of Microbiology and Immunology, Institute of Biomedicine, University of Gothenburg, Gothenburg, Sweden

**Keywords:** Epstein–Barr virus, cytomegalovirus, measles, rubella, IgG titers, plasmablasts, IL-21, immunocompetent host

## Abstract

**Background:**

Epstein–Barr virus (EBV) and cytomegalovirus (CMV) are ubiquitous and persistent herpesviruses commonly acquired during childhood. Both viruses have a significant impact on the immune system, especially through mediating the establishment of cellular immunity, which keeps these viruses under control for life. Far less is known about how these viruses influence B-cell responses.

**Objectives:**

To evaluate the impact of latent EBV and CMV infection on rubella- and measles-specific antibody responses as well as on the B-cell compartment in a prospective birth cohort followed during the first 10 years of life.

**Methods:**

IgG titers against rubella and measles vaccines were measured in plasma obtained from the same donors at 2, 5, and 10 years of age. Peripheral B-cell subsets were evaluated *ex vivo* at 2 and 5 years of age. Factors related to optimal B-cell responses including IL-21 and CXCL13 levels in plasma were measured at all-time points.

**Results:**

EBV carriage in the absence of CMV associated with an accelerated decline of rubella and measles-specific IgG levels (*p* = 0.003 and *p* = 0.019, respectively, linear mixed model analysis), while CMV carriage in the absence of EBV associated with delayed IgG decay over time for rubella (*p* = 0.034). At 5 years of age, EBV but not CMV latency associated with a lower percentage of plasmablasts, but higher IL-21 levels in the circulation.

**Conclusion:**

Our findings suggest that EBV carriage in the absence of CMV influences the B-cell compartment and the dynamics of antibody responses over time during steady state in the otherwise healthy host.

## Introduction

Human herpesviruses are widespread pathogens with a significant impact on the immune system. They are unique since they persist after primary infection in a clinically silent state known as latency. Two herpesviruses often contracted during childhood are Epstein–Barr virus (EBV), which establishes latent infection in the memory B-cell (MBC) compartment (1, 2), and Cytomegalovirus (CMV), which has a number of latency targets including CD34^+^ and CD33^+^ hematopoietic cells (3, 4). Occasional reactivation from latency into virus replicative cycles at oropharyngeal sites promotes the shedding and transmission of EBV (1, 2). CMV may be shed for months to years, mainly by children who constitute the major group that spreads this virus (5). Despite this, herpesviruses are under tight immunological control conferred primarily by CD8^+^ T-cells and NK-cells (6). Therefore, the overwhelming majority of humans carry EBV and CMV without any clinical symptoms. For long, EBV and CMV have been regarded as pathogenic and linked to autoimmunity and complications in immunosuppressed individuals, respectively (7, 8). On the other hand, a study in mice suggests that latency with EBV or CMV equivalents can be beneficial for the carrier by mediating increased protection against subsequent bacterial infections, potentially through the action of elevated systemic cytokines (9).

How herpesvirus–host interactions imprint on the immune system of otherwise healthy humans remains largely elusive, and contrary to the cellular immunity, the impact of EBV and CMV on the humoral immunity and B-cell compartment has received much less attention. Class-switched memory B cells are diminished in young EBV seropositive children (10), and both EBV and CMV have been shown to differentially influence the human B-cell repertoire (11). Likewise, we have found that acute EBV infection *in vitro* induced a redistribution between B-cell subsets, with an enrichment of CD27^+^ B-cells, in peripheral blood mononuclear cells (PBMCs) from children (12). Functional B-cell responses in EBV-infected humans might also be modulated during latency, as we earlier found that the risk of IgE-sensitization is lower in EBV carriers, if infection occurred during infancy (13, 14). Interestingly, CMV further reduced the risk of IgE-sensitization, suggesting a further modification of antibody responses associated with CMV co-infection in EBV carriers (13). In addition to IgE-responses, there are studies showing associations between herpesviruses and altered vaccine-specific IgG titers either in infants (15) or in adults (16). Collectively, these findings suggest that the humoral- and B-cell compartments may be influenced by herpesviruses. However, to the best of our knowledge, no study has investigated serological responses and B-cell subsets in relation to herpesvirus latency in the same pediatric study population with a prospective approach. To this end, we investigated the relationship between EBV and CMV serostatus and childhood vaccine-responses during the first 10 years of life and characterized the children’s B-cell subsets. Intriguingly, we show that EBV, but not CMV, latency accelerates the decay of vaccine-specific antibodies in the young immunocompetent host.

## Materials and Methods

### Study Cohort, Determination of EBV and CMV Serostatus, and Isolation of PBMCs

This study uses data from a total of *n* = 87 children (Table 1) that are part of a prospective birth cohort (*n* = 281) born between 1997 and 2000 in Stockholm, Sweden, with detailed demographic and herpesvirus serostatus data available at 2, 5, and 10 years of age (13, 14, 17). Likewise, vaccination status for the whole cohort was available at ages 3, 6, 12, 18, and 24 months, with 95 to 98% vaccine coverage at ages 18 and 24 months, respectively. The Swedish national vaccination protocol included the administration of one dose measles, mumps, and rubella (MMR) vaccine at 18 months of age, followed by a booster dose at 12 years of age for children born during the time this study cohort was formed.^1^ Subjects from whom vaccination schedule and plasma were available at *all* of the three time points were selected (*n* = 85 for rubella and *n* = 86 for measles, Table 2). Plasma IgG titers against EBV and CMV were measured, and serostatus was established at 2, 5, and 10 years of age according to previously described methods (13, 14, 17), and the carrier status and the number of children are described in Table 2. Based on this, we divided the children into three groups [EBV non-infected (i.e., both EBV^−^CMV^+^ and EBV^−^CMV^−^)], EBV-only infected (EBV^+^CMV), and co-infected (EBV^+^CMV^+^), or four groups (EBV^−^CMV^−^, EBV^−^CMV^+^, EBV^+^CMV^−^, and EBV^+^CMV^+^).

**Table 1:** Subject demographics.

Female, number/total^a^ (%)	49/86 (57.0)
Male, number/total (%)	37/86 (43.0)
Vaginal delivery, number/total (%)	74/86 (86.0)
Caesarean section, number/total (%)	12/86 (14.0)
Birth April–September, number/total (%)	46/86 (53.5)
Birth October–March, number/total (%)	40/86 (46.5)
Mother not smoking, number/total (%)	83/86 (96.5)
Father not smoking, number/total (%)	79/86 (91.9)
Older siblings, number/total (%)	47/86 (54.7)
Attending day care, number/total (%)	74/86 (86.0)
Gestation week, mean (SD, range)	39.9 (1.45, 36–43)
Birth weight, mean grams (SD, range)	3546 (510.0, 1,860–4,600)
Birth length, mean cm (SD, range)	50.3 (1.89, 45–55)
Breastfed fully, mean months (SD, range)	4.0 (1.72, 0–10)
Breastfed partially, mean months (SD, range)	9.3 (4.58, 0.75–28)
Mother’s age, mean years (SD, range)	32.2 (4.79, 22–43)
Father’s age, mean years (SD, range)	33.5 (5.23, 22–43)
Age at day care start, mean months (SD, range)	18.0 (3.08, 12–26)

*^a^Number (*n* = 86) of the same subjects followed prospectively at 2, 5, and 10 years of age with available demographic data*.

**Table 2:** Epstein–Barr virus (EBV) and cytomegalovirus (CMV) carrier status and vaccine-specific IgG titers.

A. EBV and CMV carrier status at 2, 5, and 10 years of age	

Status	Number/total^a^ (%)
EBV^+^ at 2 years	14/^§^85 (16.5)
EBV^+^ at 5 years	29/*86 (33.7)
EBV^+^ at 10 years	36/*86 (41.9)
CMV^+^ at 2 years	37/*86 (43.0)
CMV^+^ at 5 years	41/*86 (47.7)
CMV^+^ at 10 years	45/*86 (52.3)
EBV^+^CMV^+^ at 2 years	5/^ϕ^84 (6.0)
EBV^+^CMV^+^ at 5 years	12/^§^85 (14.1)
EBV^+^CMV^+^ at 10 years	18/^§^85 (21.2)

**B. Vaccine-specific IgG titers at 2, 5, and 10 years of age**	

**Specificity (plasma concentration)**	**Median (IQR, range)**

Measles IgG (mIU/ml) at 2 years	2,997 (1,628–4,620, 280–14,561)
Measles IgG (mIU/ml) at 5 years	2,186 (1,085–4,573, 150–16,232)
Measles IgG (mIU/ml) at 10 years	1,001 (407–1,732, 150–6,236)
Rubella IgG (mIU/ml) at 2 years	87 (61–140, 21–214)
Rubella IgG (mIU/ml) at 5 years	57 (37–103, 7–196)
Rubella IgG (mIU/ml) at 10 years	37 (16–59, 4–189)

^a^Number (*n* = 87) of the same subjects followed prospectively at 2, 5, and 10 years of age. Data missing from ^§^one, ^ϕ^two, or *three donors.

*IQR, interquartile range*.

Peripheral blood mononuclear cells were separated by Ficoll–Hypaque gradient centrifugation (GE Healthcare Bio-Sciences AB, Uppsala, Sweden). The cells were washed and re-suspended in freezing medium consisting of 50% FCS (Gibco, Invitrogen, Carlsbad, CA, USA), 10% dimethyl sulfoxide (Sigma Aldrich, St Louis, MO, USA) and 40% RPMI-1640, and stored in liquid nitrogen until further analysis. PBMCs were available from 2-year olds (*n* = 45) and 5-year olds (*n* = 35). The study was approved by the Human Ethics Committee at Huddinge University Hospital, Stockholm (Dnr 75/97, 331/02, 2007/858-31/2). In agreement with the Human Ethics Committee and according to the regulations at the time of the initiation of the study, the parents provided either their informed verbal (age 2 and 5 years) or written (age 10 years) consent.

### Determination of B-Cell and T-Follicular Helper (T_FH_) Cell Subset Phenotypes by Flow Cytometry

Peripheral blood mononuclear cells from 2- and 5-year olds were stained *ex vivo* with alternating combinations of: CD19, CD27, CD38, IgD (from BD Biosciences, San Jose, CA, USA) to characterize B-cell populations. For identification of cells with a T_FH_-phenotype in 2-year olds, CD3, CD4, CD45RO, PD-1, and CXCR5 (BD Biosciences) were used. A fixable dead cell marker (Invitrogen) was used to exclude nonviable cells from the analysis. The FACSVerse or LSR II flow cytometers were used to acquire data (BD Biosciences) and FlowJo Software (TreeStar, Ashland, OR, USA) was used to analyze the data. Lymphocytes were gated based on forward scatter and side scatter properties, followed by gating of B cells (CD19^+^ lymphocytes), which were further defined as MBCs by the expression of surface CD27. Alternatively, B-cells were classified into four populations based on the expression of CD27 and IgD as follows: CD27^−^IgD^+^ (naive) B-cells, CD27^−^IgD^−^ [double negative (DN)], CD27^+^IgD^+^, and CD27^+^IgD^−^ B-cells according to the established classification system for MBCs (18). Plasmablasts were identified as CD27^high^CD38^high^ B-cells. B-cell gating strategies are displayed in Figure S1A in Supplementary Material. For examination of T_FH_-cells, CD3^+^CD4^+^ lymphocytes were assessed for the simultaneous expression of CXCR5, PD-1, and CD45RO (Figure S1B in Supplementary Material). FMO and isotype controls were used for appropriate gating of CXCR5 and PD-1, respectively.

### Quantification of Measles and Rubella Specific-IgG Titers, Enzyme-Linked Immunosorbent (ELISA), and Luminex Assay

Measles and rubella-specific IgG concentrations were assessed by Enzygnost^®^ anti-measles IgG (Dade Behring, Marburg, GmbH) and Platelia™ Rubella IgG ELISA kits (Bio-Rad Laboratories, Hercules, CA, USA), respectively, according to the manufacturer’s instructions. The protective titer cutoff for measles-specific IgG was >150 mIU/ml and that for rubella was >10 IU/ml as per manufacturer manuals. The levels of IL-21 in plasma were measured using the human IL-21 ELISA development kit (MabTech AB, Stockholm, Sweden), according to the instructions from the manufacturer. The plasma CXCL13 chemokine levels were detected using the Bio-Plex Pro Human Chemokine 7-plex kit (Bio-Rad, USA), according to the manufacturer’s instructions.

### Statistics

The distribution of measles and rubella IgG titers was visually inspected and log-transformed using the natural logarithm to better fulfill the assumptions of linearity and homoscedasticity of the assessed models. For valid measurements of measles and rubella IgG at 2, 5, and 10 years of age, random intercept and random slope linear mixed models were fitted. Age was added as a random effect per child, and as a fixed effect. Latency with EBV and CMV were added as fixed effects. Likelihood-ratio and Wald tests were used to assess significance of covariates. The models were fitted using lmer in the lme4 package (19) in R version 3.2.5.^2^ Mann–Whitney *U*-test was used to test statistical differences between the EBV-only infected group and the EBV non-infected or the co-infected groups. *p*-values < 0.05 were considered statistically significant. All dot plots show median (horizontal middle lines) and interquartile range (whiskers). GraphPad prism 7.02 (GraphPad, La Jolla, CA, USA) was used for these statistical analyses.

## Results

### EBV Latency Associates with an Accelerated Decay of Vaccine-Specific Antibody Titers

**Figure 1:**
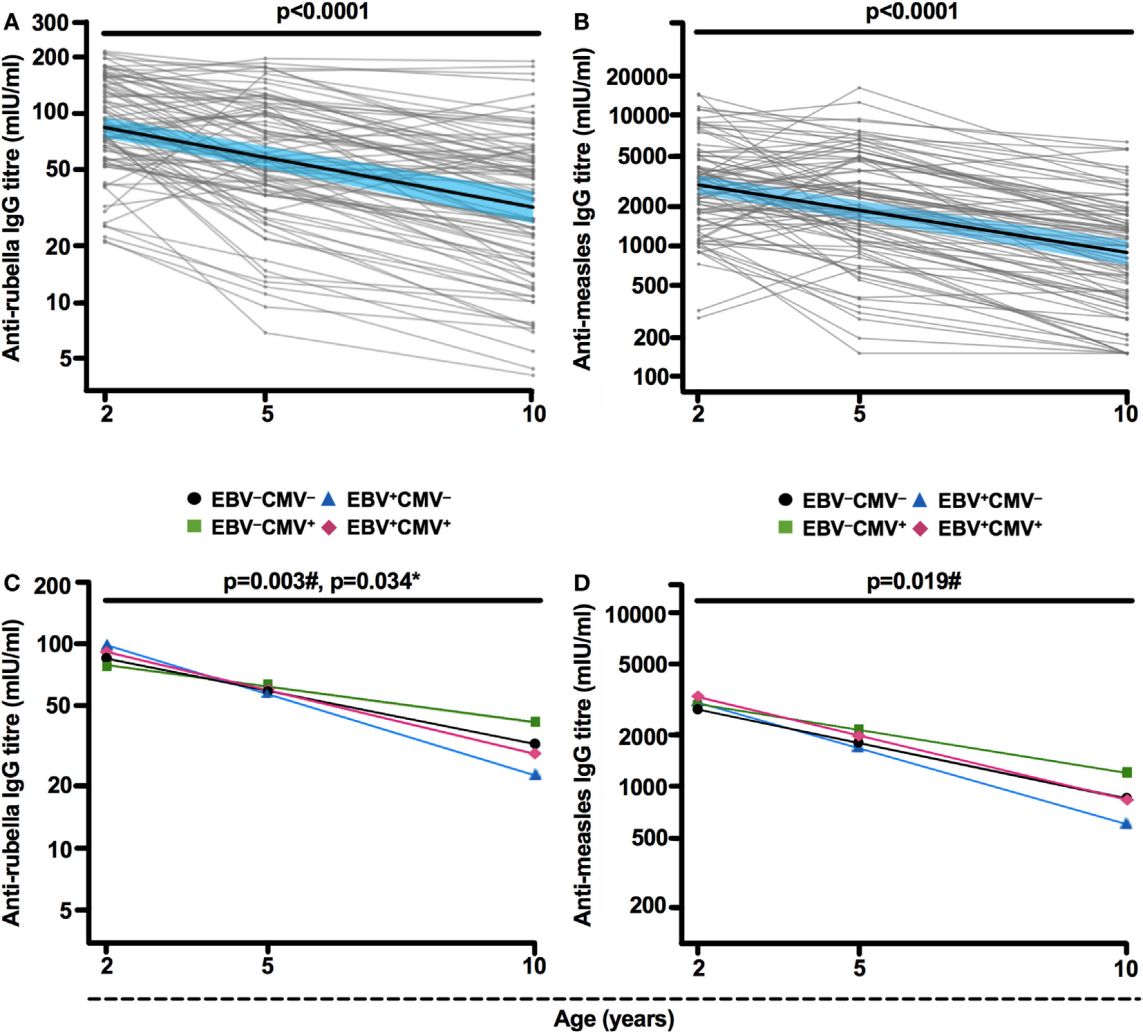
Epstein–Barr virus (EBV) latency associates with an accelerated decay of vaccine-specific IgG titers. **(A)** Anti-rubella and **(B)** anti-measles IgG titers at 2, 5, and 10 years of age. Thick black lines show mean IgG titers over time, along with 95% Wald confidence intervals in blue. Thin gray lines depict **(A)** rubella-specific (*n* = 85) and **(B)** measles-specific (*n* = 86) IgG titers for each included individual. Mean **(C)** anti-rubella and **(D)** anti-measles IgG titer change over time based on EBV and cytomegalovirus (CMV) carrier status resulting in four groups: EBV^−^CMV^−^ (black slope), EBV^+^CMV^−^ (blue slope), EBV^−^CMV^+^ (green slope), and EBV^+^CMV^+^ (red slope). ^#^*p*-values or *p-values were generated after comparison of EBV^+^CMV^−^ or EBV^−^CMV^+^, respectively, with the EBV^−^CMV^−^ (reference) group by linear mixed model analysis.

To examine whether latent EBV infection influences vaccine responses, we analyzed anti-rubella and anti-measles IgG titers over time in children with different EBV and CMV serostatus. We used a linear-mixed effect model approach, which considers the fact that the children will seroconvert at different time points. In accordance with current knowledge (20), the vaccine-specific titers declined significantly over time, with an average decrease of 0.12 and 0.15 in IgG titer per year for rubella and measles, respectively (*p* < 0.0001 for both, Figures 1A,B). Interestingly, the EBV^+^CMV^−^ group showed a significantly more rapid reduction of vaccine-specific titers compared to the EBV^−^CMV^−^ group (*p* = 0.003 and *p* = 0.019 for rubella and measles, respectively, Figures 1C,D). In contrast, the EBV^−^CMV^+^ group had a significantly slower decrease in rubella-specific titers compared to the EBV^−^CMV^−^ group (*p* = 0.034, Figure 1C), while the halted decrease in measles-specific titers was observable but not statistically significant (*p* = 0.12, Figure 1D). Notably, the pattern of antibody decay in the co-infected (EBV^+^CMV^+^) group was similar to the reference (EBV^−^CMV^−^) group (Figures 1C,D). Three-way interactions for age, EBV status, and CMV status were found non-significant (*p* = 0.26 and *p* = 0.94 for measles and rubella, respectively), indicating that it is sufficient to model the virus-associated effects in an additive fashion. In contrast, total IgG levels in plasma did not differ between the groups (data not shown).

### The Proportions of Naive and Memory B-Cells Alter with Age but Are Not Further Influenced by EBV

**Figure 2:**
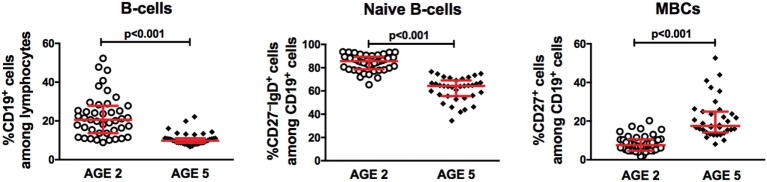
Changes in the distribution of peripheral B-lymphocyte subsets with age. Flow cytometry analysis of peripheral blood mononuclear cells (PBMCs) from children at 2 (*n* = 43) and 5 years of age (*n* = 35) for proportions of total B-cells (CD19^+^ cells among lymphocytes), naive B-cells (CD27^−^IgD^+^ cells among CD19^+^ lymphocytes), and memory B-cells (MBCs) (CD27^+^ cells among CD19^+^ lymphocytes).

Since EBV infection was found to be associated with changes in serological immunity during childhood, we investigated whether EBV also imprinted the B-cell compartment and characterized the B-cells in the 2- and 5-year-old children. As expected, we observed decreased proportions of total and naive B-cells, but increased proportions of MBCs, with age (all *p* < 0.001) (Figure 2).

**Figure 3:**
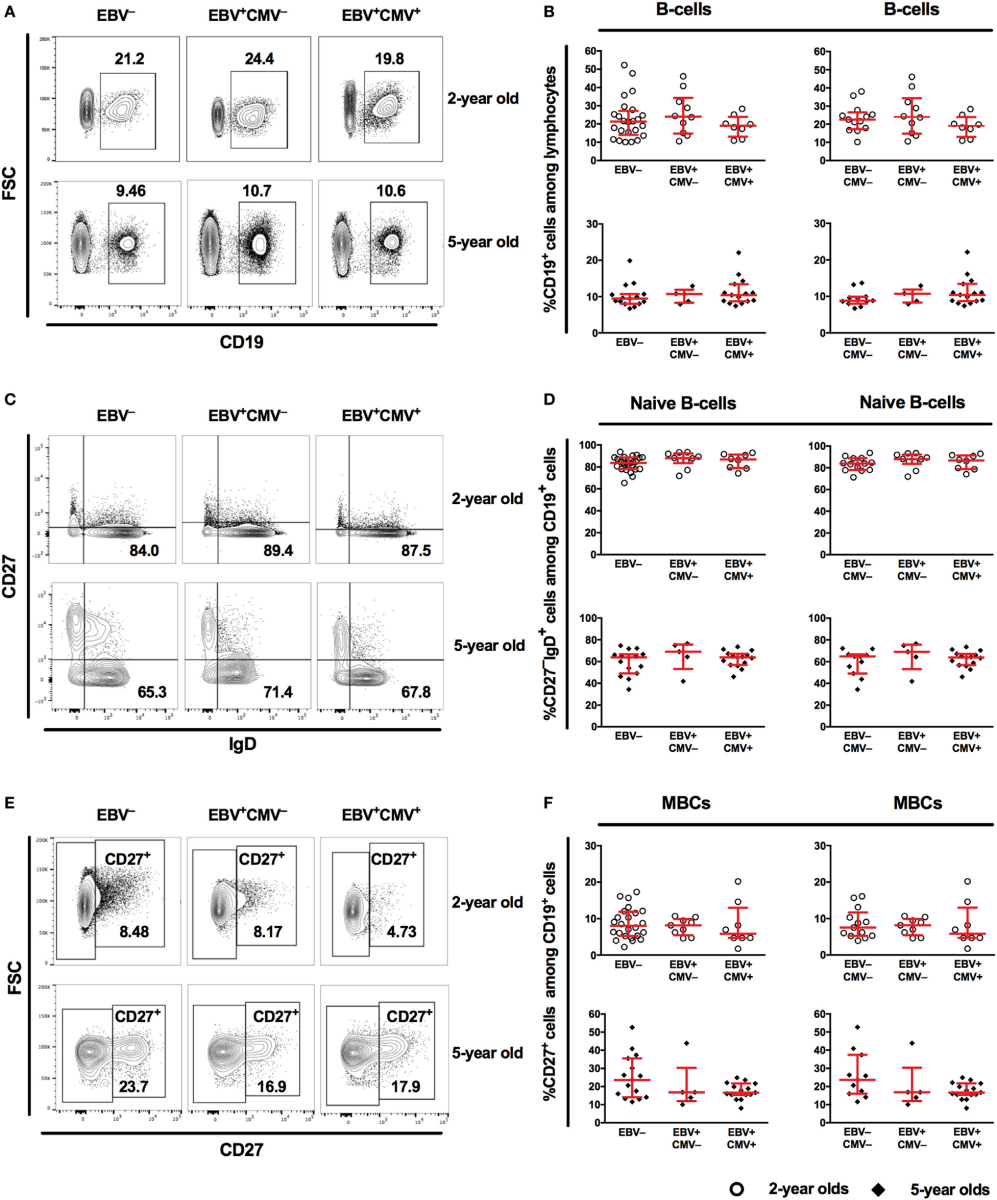
Characteristics of the peripheral B-cell compartment in relation to Epstein–Barr virus (EBV) carriage at 2 and 5 years of age. **(A)** Representative gating and **(B)** combined data for the proportions of CD19^+^ cells among lymphocytes from EBV^−^, EBV^+^CMV^−^ or EBV^+^CMV^+^ donors. **(B)** Right panel shows exclusion of CMV^+^ donors from the EBV^−^ group. **(C)** Representative gating and **(D)** combined data for the proportions of naive B-cells among CD19^+^ cells in EBV^−^, EBV^+^CMV^−^, or EBV^+^CMV^+^ donors. **(D)** Right panel shows exclusion of CMV^+^ donors from the EBV^−^ group. **(E)** Representative gating and **(F)** combined data for the proportions of memory B-cells (MBCs) among CD19^+^ cells in EBV^−^, EBV^+^CMV^−^, or EBV^+^CMV^+^ donors. **(F)** Right panel shows exclusion of CMV^+^ donors from the EBV^−^ group. At age 2 years: EBV^−^ [*n* = 25 in **(A,C,E)** and *n* = 13 in **(B,D,F)**], EBV^+^CMV^−^ (*n* = 10 in all graphs), and EBV^+^CMV^+^ (*n* = 10 in all graphs.) At age 5 years: EBV^−^ [*n* = 15 in **(A,C,E)** and *n* = 11 in **(B,D,F)**], EBV^+^CMV^−^ (*n* = 5 in all graphs), and EBV^+^CMV^+^ (*n* = 15 in all graphs).

**Figure 4:**
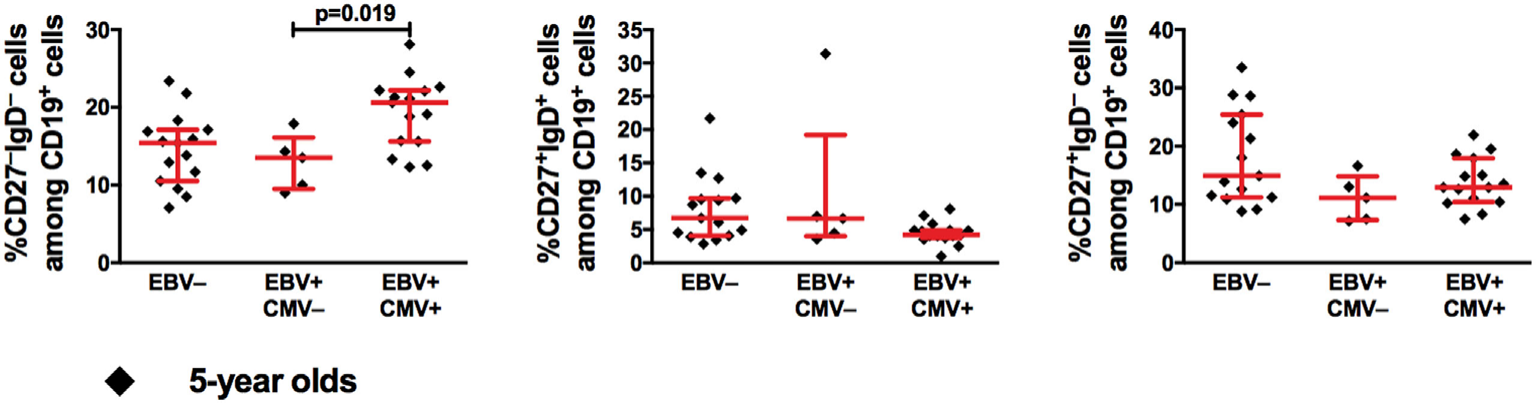
Epstein–Barr virus (EBV) and cytomegalovirus (CMV) co-infection is associated with higher proportions of CD27^−^IgD^−^ B-cells at 5 years of age. From left to right, the proportions of CD27^−^IgD^−^, CD27^+^IgD^+^, and CD27^+^IgD^−^ cells among CD19^+^ cells. EBV^−^ (*n* = 15), EBV^+^CMV^−^ (*n* = 5), and EBV^+^CMV^+^ (*n* = 15).

We then investigated the B-cell compartment in relation to EBV serostatus comparing the EBV-only infected (EBV^+^CMV^−^) to either EBV non-infected (EBV^−^) or co-infected (EBV^+^CMV^+^) groups. At 2 and 5 years of age, there were no significant differences in the percentages of total B-cells, naive B-cells, or MBCs between the groups (Figures 3A–F). Exclusion of the CMV^+^ donors from the EBV non-infected group did not alter these results (Figures 3B,D,F right panels). When further characterizing the peripheral B-cells according to their CD27- and IgD-expression, we found a significant accumulation of CD27^−^IgD^−^ cells (DN MBCs) in the co-infected group (*p* = 0.019); however, there were no differences in IgG, IgA, or IgM expression within this sub-population (data not shown). Further, there were no differences in the CD27^+^IgD^+^ and CD27^+^IgD^−^ B-cells at 5 years of age (Figure 4).

### EBV-Only Infected 5-Year-Old Children Show Lower Proportions of Antibody-Producing Cell Precursors

**Figure 5:**
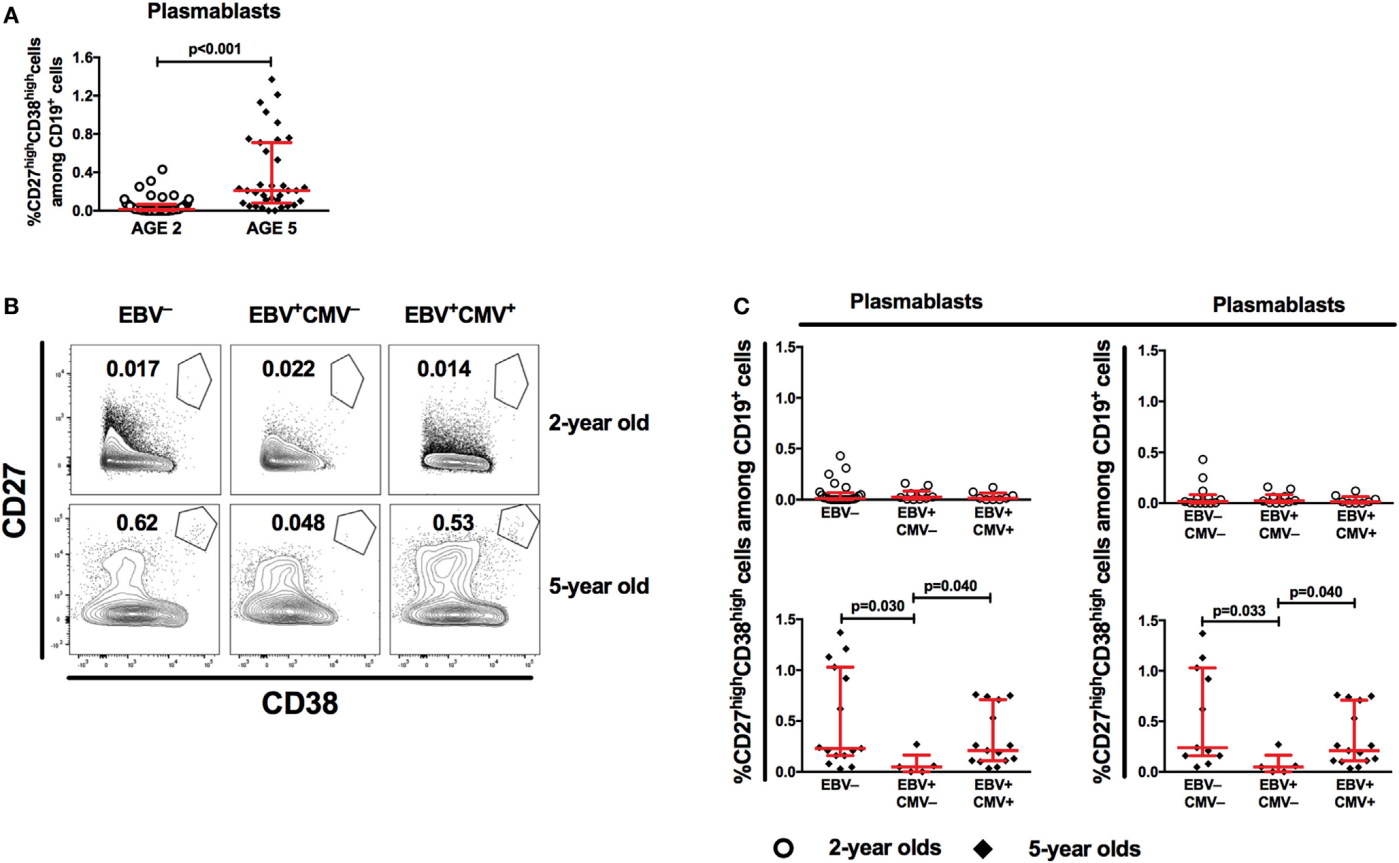
Epstein–Barr virus (EBV) carriers show altered proportions of peripheral antibody-producing cell precursors at 5 years of age. **(A)** Flow cytometry analysis of peripheral blood mononuclear cells (PBMCs) from children at 2 years (*n* = 43) and 5 years of age (*n* = 35) for proportions of plasmablasts (CD27^high^CD38^high^ cells among CD19^+^ cells). **(B)** Representative gating of plasmablasts and **(C)** combined data for plasmablast proportions in EBV^−^, EBV^+^CMV^−^, or EBV^+^CMV^+^ donors at 2 and 5 years of age. **(C)** Right panel shows the exclusion of CMV^+^ donors from the EBV^−^ group. At age 2 years: EBV^−^ [*n* = 25 in **(B)** and *n* = 13 in **(C)**], EBV^+^CMV^−^ [*n* = 10 in both **(B,C)**], and EBV^+^CMV^+^ [*n* = 10 in both **(B,C)**]. At age 5 years: EBV^−^ [*n* = 15 in **(B)** and *n* = 11 in **(C)**], EBV^+^CMV^−^ [*n* = 5 in both **(B,C)**], and EBV^+^CMV^+^ [*n* = 15 in both **(B,C)**].

Based on the strong association between EBV infection and the accelerated loss of protective antibodies against both rubella and measles, we further investigated the percentage of plasmablasts, which are peripheral markers of serological immune responses (21). As expected, the percentage of peripheral plasmablasts increased with age (Figure 5A). Further, we found a significantly lower proportion of plasmablasts in the EBV-only infected group at 5 years of age (Figures 5B,C), which was sustained following exclusion of CMV^+^ donors from the EBV non-infected group (Figure 5C right panel). These differences could not be observed at 2 years of age (Figures 5B,C).

### EBV and CMV Latency Differentially Relate to Plasma IL-21 Levels

T_FH_-cells are dedicated to contribute to B-cell responses in secondary-lymphoid tissues, where long-lived plasma cell precursors and MBCs with high-affinity antibodies are developed. One central factor for the T_FH_-cell mediated generation of B-cell responses is IL-21 (22). In addition, CXCL13 is a chemokine important for the proper positioning of CXCR5^+^ cells (e.g., B- and T_FH_-cells) in the secondary-lymphoid tissues and alterations in plasma CXCL13 have been connected to immune function (23).

**Figure 6:**
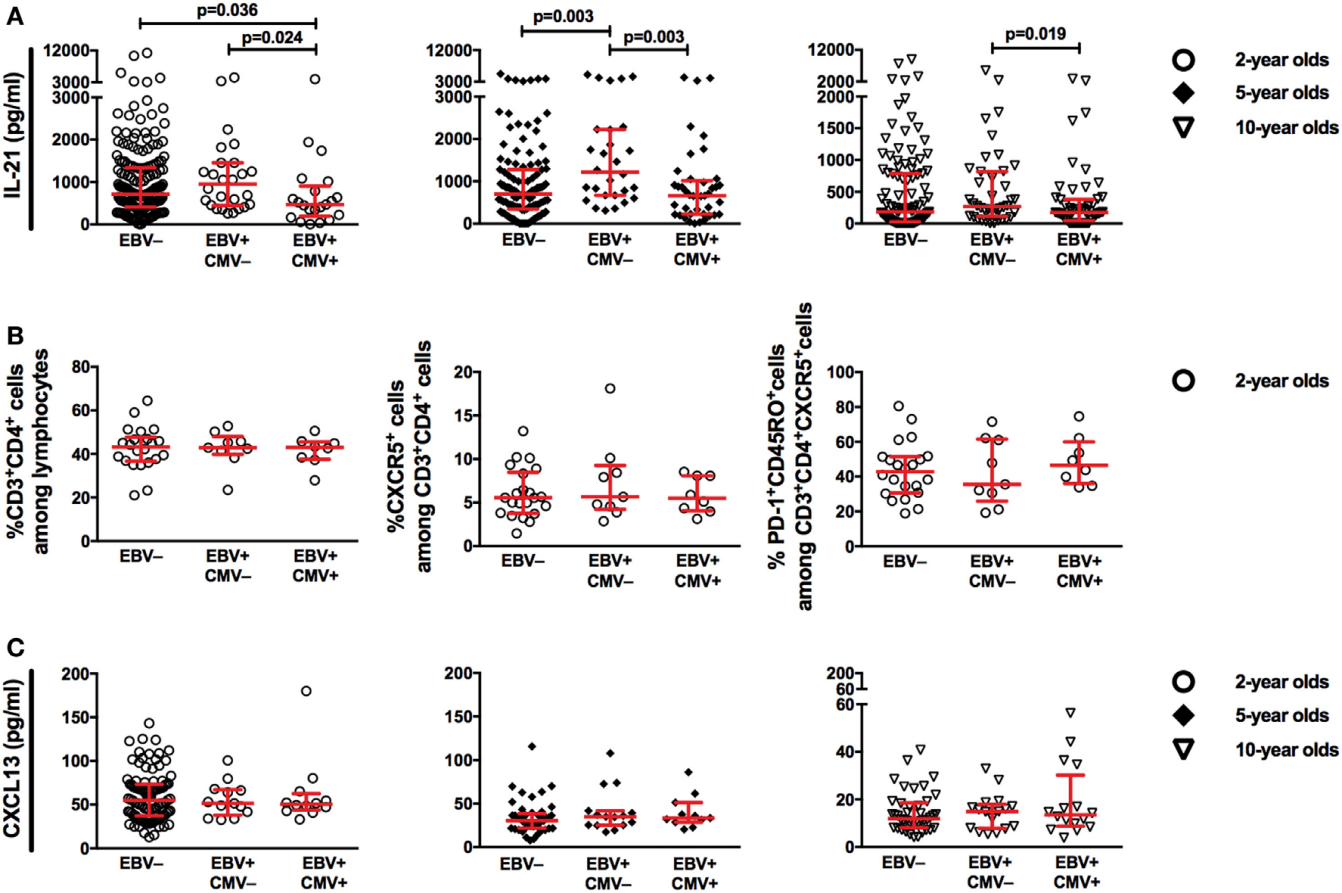
Epstein–Barr virus (EBV), but not cytomegalovirus (CMV), infection is associated with higher plasma IL-21 during childhood. **(A)** Plasma IL-21 levels at 2, 5, and 10 years of age, **(B)** the proportions of CD3^+^CD4^+^ T-cells among lymphocytes, CXCR5^+^ cells among CD3^+^CD4^+^ T-cells and PD-1^+^CD45RO^+^ cells among CD3^+^CD4^+^CXCR5^+^ T-cells at 2 years of age, **(C)** plasma CXCL13 levels at 2, 5, and 10 years of age, in relation to EBV and CMV serostatus. **(A)** EBV^−^ (*n* = 189, *n* = 118, and *n* = 111), EBV^+^CMV^−^ (*n* = 26, *n* = 28, and *n* = 42), and EBV^+^CMV^+^ (*n* = 21, *n* = 41, and *n* = 53) at 2, 5, and 10 years of age, respectively. **(B)** EBV^−^ (*n* = 22), EBV^+^CMV^−^ (*n* = 9), or EBV^+^CMV^+^ (*n* = 8) in 2-year-old donors. **(C)** EBV^−^ (*n* = 98, *n* = 45, and *n* = 42), EBV^+^CMV^−^ (*n* = 13, *n* = 15, and *n* = 17), and EBV^+^CMV^+^ (*n* = 12, *n* = 11, and *n* = 16) at 2, 5, and 10 years of age, respectively.

We started by examining the relationship between EBV latency and IL-21 in the circulation of the children. Interestingly, at 2 and 5 years of age, EBV^+^CMV^−^ donors had higher plasma levels of IL-21 compared to the EBV non-infected and co-infected groups (Figure 6A). This pattern was less obvious by the age of 10 years but remained significant between the EBV-only and co-infected group. However, we found no alteration in the percentage of circulating CD4^+^ T-cells, CXCR5^+^ CD4^+^ T-cells, or T-cells with a T_FH_-phenotype in the EBV^+^CMV^−^ group at 2 years of age where PBMCs were available (Figure 6B). Further, there were no differences found in plasma CXCL13 levels at either of the examined time points (Figure 6C). Exclusion of the CMV^+^ donors from the EBV non-infected group did not alter these results (Figure S2 in Supplementary Material).

## Discussion

Herein, we used a prospective approach to address whether the latency with two common herpesviruses, which are usually contracted in childhood, could influence B-cell vaccine responses in a young and otherwise healthy population. To do this, we measured IgG titers against childhood vaccines as our study model in a well-characterized prospective Swedish birth cohort (13, 14, 17) with high vaccination coverage. In line with current agreement of prime-boost strategy for MMR vaccination (24), we show that measles and rubella-specific antibody responses decay over time following the first vaccine dose. Interestingly, EBV, but not CMV, latency was associated with a more rapid decline of measles and rubella-specific IgG titers. Further, we show that antibody responses in EBV and CMV co-infected children were comparable to the EBV and CMV non-infected group for both measles and rubella antigens, suggesting that CMV to some extent halts, restores, or compensates EBV-associated vaccine-antibody titer loss. In consistence with our results, infection with EBV, but not CMV, associated with lower antibody responses to measles vaccine-antigen in Gambian infants (15). Two recent studies using material from the same cohort of children showed no differences in vaccine responses against measles antigen at 6 years of age in relation to EBV and/or CMV-infection (10, 25). In the first study, children were divided into EBV non-infected, early EBV-infected (<14 months) and late EBV-infected (14 months to 6 years) groups (10), while in the second study, the children were divided into four groups based on both EBV and CMV serostatus (25). However, both of these studies lack a longitudinal approach for the IgG measurements that specifically addresses how infection with herpesviruses over several years may affect a given individual’s serological response. In our study, by investigating a relatively large group where each child was followed prospectively, we were able to account for longitudinal effects of herpesviruses on humoral immunity.

To the best of our knowledge, we are the first to report that EBV and CMV associate with opposite effects on IgG responses to rubella and measles vaccines. Albeit not studying the same immunogens, our results are in agreement with a recent study where CMV seropositive healthy young adults had significantly higher influenza-specific antibody responses compared to their seronegative peers (16). Why EBV and CMV have an opposite effect on the serological memory compartment of young individuals is puzzling. However, immune responses against CMV and EBV are qualitatively different in many ways. For instance, CMV provokes a T_H_1 cytokine-biased response, while EBV has been associated with a mixed T_H_1 and T_H_2-cell cytokine profile in children (26–28). Thus, we could speculate that EBV and CMV may promote the expansion of memory cell populations that differently shape ongoing or *de novo* B-cell responses. However, a recent study was unable to report an association between enriched CD8^+^ memory T-cells and measles IgG titers in EBV- or CMV-infected children (25). Yet, that study differed from ours with respect to sampling occasions and follow-up time as well as applying a cross-sectional approach for their analysis (25).

Albeit there are overwhelming data on B-cell infection biology and phenotypes upon EBV infection, the research focus has been on models with a significantly modulated immune system such as cancers and autoimmunity (29, 30) or during acute EBV infection in adolescence and adulthood (31–34). A general consensus from these studies is that the EBV reservoir is confined within the IgD^−^CD27^+^ MBCs. Another study focusing on otherwise healthy young patients, IgD^+^ B-cells in tonsillar biopsies were identified as EBV latency-reservoirs (35).

In order to understand whether the influence of EBV and CMV on vaccine responses could be linked to the cellular compartment of B-cells in our cohort of healthy children, we characterized and followed the dynamics of these cells at 2 and 5 years of age where PBMC samples were available. We found that the proportions of MBCs increased with age, indicating a higher output of cells derived from lymphoid tissues. Furthermore, compared to their non- or single-virus infected peers, EBV and CMV co-infected children had markedly higher proportions of DN MBCs at 5 years of age. Indeed, this sub-population has been associated with a chronic immune activation and may be the result of long-lasting antigenic load as seen in elderly (36). Interestingly, previously we also found an enrichment of terminally differentiated NK-cells, which are markers of chronic immune activation, in this group of children (37). How persistence of DN MBC, or other cells with a phenotype associated with chronic activation, shape humoral immunity in children remains to be determined.

Vaccine-specific antibody levels are maintained by terminally differentiated, non-proliferating, long-lived plasma cells, which originate from the late stages of germinal center reactions occurring in secondary lymphoid tissues (38–40). Following a typical germinal center response, plasmablasts migrate to the bone marrow to complete their differentiation into plasma cells. We found the percentage of plasmablasts in PBMCs to be low at the age of 2 years, regardless of herpesvirus infection status. A general rise in the proportions of plasmablasts was however noted among 5-year-old children except in the EBV^+^CMV^−^ group, which had markedly lower percentages compared to the remaining groups. It is unlikely that these plasma cell precursors have antigen-specificity for vaccines administered years before. However, it has been shown that the replicative cycle of EBV is induced, while cells are terminally differentiating into plasma cells *in vivo* (32, 41). Thus, a lower circulating plasmablast proportion could hypothetically be a marker of altered germinal center output. In light of the above, EBV was also shown to be responsible for altered expression of chemokine receptors important for proper positioning of B-cells during germinal center reactions (42). This could negatively affect plasmablast generation, further supporting the idea that EBV could diminish plasmablast numbers and/or germinal center output.

IL-21, mainly produced by T_FH_-cells, but also by others including T-follicular regulatory and T_H_17-cells, is a key cytokine required for successful germinal center reactions and generation of antibody-secreting cells (22, 43–46). In addition, the CXCL13-CXCR5 signaling axis is central in proper organization of B-cell follicles and germinal centers (23). Therefore, we aimed to examine IL-21, CXCL13, and T_FH_-cells in order to understand the relationship between these factors and changes in IgG responses in EBV-infected children. Surprisingly, IL-21 plasma levels were significantly higher in the EBV^+^CMV^−^ group at all-time points. The reason behind this is not clear. Interestingly, however, the treatment of EBV-infected cells and cell lines with IL-21 *in vitro* aids in the maintenance of viral latency through induction of EBV latent membrane protein 1 (LMP1) (47, 48). LMP1-expressing B-cells halt germinal center-formation and induce the extrafollicular B-cell differentiation pathway (49), which is related to short-lived antibody responses and might reflect antibody-response decay over time. Although we cannot exclude the possibility that herpesviruses influence the T_FH_-compartment later in childhood, the systemic IL-21 pattern was not paralleled by the proportions of T_FH_-cells in 2-year-old children. Neither did the total numbers of IL-21 secreting PBMCs, measured by ELISPOT upon T-cell receptor stimulation (data not shown) differ between the groups, suggesting an alternative source of IL-21 in the circulation. However, due to the limited number of cells available from the children, we could not investigate this possibility.

Some limitations should be acknowledged. The availability of PBMC samples from the same donors was limited. In this regard, it was not possible to perform further analyses of circulating B-cell antigen specificity, which could contribute to better associations between serological profiles and examined B-cell subsets. Likewise, a more thorough analysis of the T_FH_ compartment was not feasible.

Whether an accelerated decay of vaccine-specific IgG levels in CMV-naive EBV-infected children is significant enough to negatively impact the protection against measles and/or rubella remains unknown due to the fact that measles and/or rubella outbreaks in Sweden are extremely rare. Yet, this warrants further investigations if EBV- and CMV seroprevalence will change in the general population; something that is indicated in this particular cohort (17).

In conclusion, we believe that our study is unique due to its longitudinal approach, showing antibody responses to measles and rubella vaccine antigens at three occasions during the first 10 years of life in relation to herpesvirus carriage. These observations along with analysis of the B-cell compartment during infancy and childhood contributes to a better understanding of how B-cell responses to certain antigens are affected by herpesvirus carriage during steady state.

## Ethics Statement

The study was approved by the Human Ethics Committee at Huddinge University Hospital, Stockholm (Dnr 75/97, 331/02, 2007/858-31/2). In agreement with the Human Ethics Committee and according to the regulations at the time of the initiation of the study, the parents provided either their informed verbal (age 2 and 5 years) or written (age 10 years) consent.

## Author Contributions

MB, ES-E, SS-H, and AN conceived and designed the study. GL, SB, CC-Q, BN, and MB designed and/or performed lab work. GL, SB, IM, AN, SS-H, and ES-E analyzed and/or finalized the data. GL and IM performed statistical analysis. CN performed clinical evaluation and follow-up of the children. GL, SB, SS-H, and ES-E wrote the paper. All authors read and approved the final version of the manuscript.

## Conflict of Interest Statement

The authors declare that the research was conducted in the absence of any commercial or financial relationships that could be construed as a potential conflict of interest.

## Footnotes

^1^http://sammantradeshandlingar.sll.se/sites/sammantradeshandlingar.sll.se/Handlingar/HSN/2007/2007-03-22/08mBILAGA1.pdf.

^2^http://www.gbif.org/resource/81287.

## Abbreviations

EBV, Epstein–Barr virus; CMV, cytomegalovirus; T_H_-cells, T-helper cells; T_FH_-cells, T-follicular helper cells; Ig, immunoglobulin; MBCs, memory B-cells.
