# Supplementary material for: Epstein–Barr Virus, but Not Cytomegalovirus, Latency Accelerates the Decay of Childhood Measles and Rubella Vaccine Responses—A 10-Year Follow-up of a Swedish Birth Cohort
Source: Front Immunol. 2017 Dec 21;8:1865. doi: 10.3389/fimmu.2017.01865 (PMC5742589; doi:10.3389/fimmu.2017.01865)
Supplement: Supplementary file 1 [file image_1.PDF]

SUPPLEMENTARY FIGURE 1.

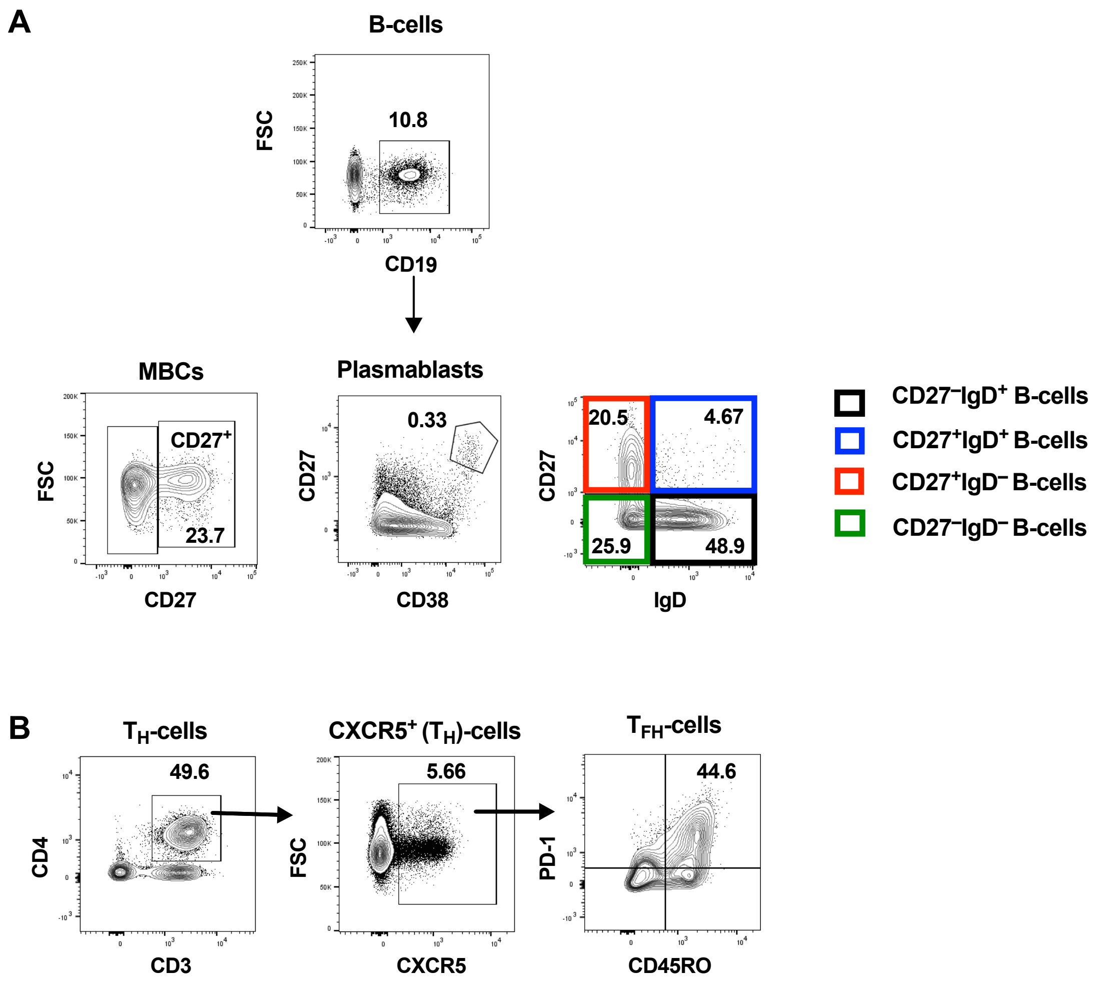

Supplementary Figure 1. Gating strategies for analysis of B-cell subsets and T_{FH}-cells. **(A)** B-cells were defined as $CD19^+$ lymphocytes, which were further classified into MBCs ($CD27^+$ cells) and plasmablasts ($CD27^{high}CD38^{high}$ cells). IgD^+CD27^- B-cells (black quadrant), IgD^+CD27^+ B-cells (blue quadrant), IgD^-CD27^+ B-cells (red quadrant) and IgD^-CD27^- B-cells (green quadrant) were gated based on their CD27 and IgD expression among $CD19^+$ lymphocytes. **(B)** Representative gating of T_{FH}-cells defined as PD-$1^+CD45RO^+$ cells among the $CXCR5^+CD4^+CD3^+$ T-lymphocyte population.
