# Supplementary material for: Epstein–Barr Virus, but Not Cytomegalovirus, Latency Accelerates the Decay of Childhood Measles and Rubella Vaccine Responses—A 10-Year Follow-up of a Swedish Birth Cohort
Source: Front Immunol. 2017 Dec 21;8:1865. doi: 10.3389/fimmu.2017.01865 (PMC5742589; doi:10.3389/fimmu.2017.01865)
Supplement: Supplementary file 2 [file image_2.PDF]

SUPPLEMENTARY FIGURE 2.

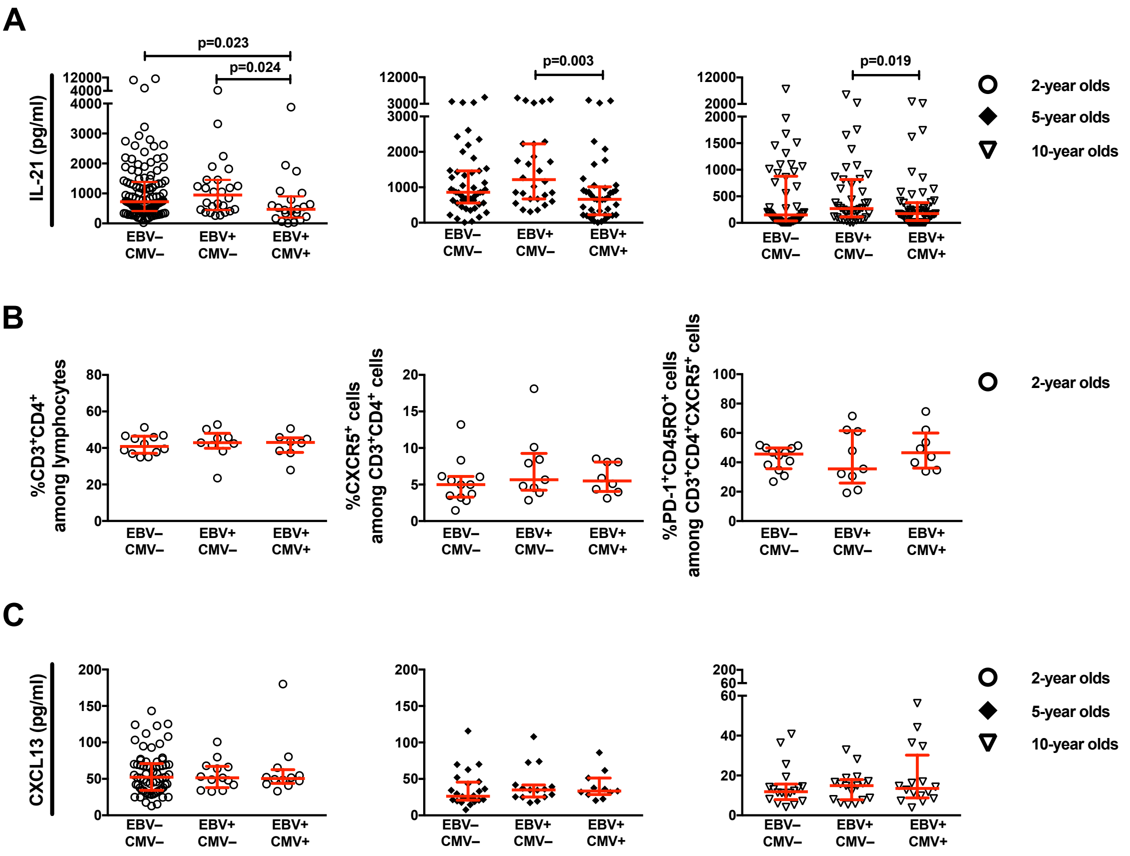

Supplementary Figure 2. CMV$^+$ donors were excluded from the EBV non-infected group. **(A)** Plasma IL-21 levels at 2-, 5- and 10-years of age, **(B)** the proportions of CD3$^+$CD4$^+$ T-cells among lymphocytes, CXCR5$^+$ cells among CD3$^+$CD4$^+$ T-cells and PD-1$^+$CD45RO$^+$ cells among CD3$^+$CD4$^+$CXCR5$^+$ T-cells at 2-years of age, **(C)** plasma CXCL13 levels at 2-, 5- and 10-years of age, in relation to EBV and CMV serostatus.

At age 2 years: EBV$^-$CMV$^-$ (n=119 in **A**, n=12 in **B** and n=66 in **C**), EBV$^+$CMV$^-$ (n=26 in **A**, n=9 in **B** and n=13 in **C**) and EBV$^+$CMV$^+$ (n=21 in **A**, n=8 in **B** and n=12 in **C**). At age 5 years: EBV$^-$CMV$^-$ (n= 51 in **A** and n=24 in **C**), EBV$^+$CMV$^-$ (n=28 in **A** and n=15 in **C**) and EBV$^+$CMV$^+$ (n=40 in **A** and n=11 in **C**). At age 10 years: EBV$^-$CMV$^-$ (n=50 in **A** and n=18 in **C**), EBV$^+$CMV$^-$ (n=42 in **A** and n=17 in **C**) and EBV$^+$CMV$^+$ (n=53 in **A** and n=16 in **C**).
